# Workplace Exposures Vary Across Neighborhoods in the US: Implications on Social Vulnerability and Racial/Ethnic Health Disparities

**DOI:** 10.1007/s40615-024-02143-5

**Published:** 2024-08-30

**Authors:** Abas Shkembi, Jon Zelner, Sung Kyun Park, Richard Neitzel

**Affiliations:** 1 Department of Environmental Health Sciences, University of Michigan School of Public Health, Ann Arbor, MI 48109 USA; 2 Department of Epidemiology, University of Michigan School of Public Health, Ann Arbor, MI USA; 3 Center for Social Epidemiology and Population Health, University of Michigan School of Public Health, Ann Arbor, MI USA

**Keywords:** Occupational justice, Social vulnerability index, Disparities, Environmental justice, Social determinants of health

## Abstract

Ignoring workplace exposures that occur beyond the local residential context in place-based risk indices like the CDC’s Social Vulnerability Index (SVI) likely misclassifies community exposure by under-counting risks and obscuring true drivers of racial/ethnic health disparities. To investigate this hypothesis, we developed several place-based indicators of occupational exposure and examined their relationships with race/ethnicity, SVI, and health inequities. We used publicly available job exposure matrices and employment estimates from the United States (US) Census to create and map six indicators of occupational hazards for every census tract in the US. We characterized census tracts with high workplace–low SVI scores. We used natural cubic splines to examine tract level associations between the percentage of racial/ethnic minorities (individuals who are not non-Hispanic White) and the occupational indicators. Lastly, we stratified each census tract into high/low occupational noise, chemical pollutant, and disease/infection exposure to examine racial/ethnic health disparities to diabetes, asthma, and high blood pressure, respectively, as a consequence of occupational exposure inequities. Our results show that racial/ethnic minority communities, particularly those that are also low-income, experience a disproportionate burden of workplace exposures that may be contributing to racial/ethnic health disparities. When composite risk measures, such as SVI, are calculated using only information from the local residential neighborhood, they may systematically under-count occupational risks experienced by the most vulnerable communities. There is a need to consider the role of occupational justice on nationwide, racial/ethnic health disparities.

**Supplementary Information:**

The online version contains supplementary material available at 10.1007/s40615-024-02143-5.

## Introduction

The workplace plays an important role shaping patterns of exposure to a wide range of environmental, infectious, and social risks that influence the health and economic burden of communities. Workplace hazards (e.g., chemical exposures, musculoskeletal disorders, COVID-19, psychosocial strain and stress) are important causes of chronic disease, debilitating injury, permanent disability, and death. Globally, work-related diseases and injuries are directly responsible for over 1.9 million deaths annually [1], not considering the burden of acute and chronic injuries and illnesses workers suffer from every day. Given an individual’s job often influences their socioeconomic status and their access to healthcare, workplaces shape the ability to overcome financial hardships resulting from these workplace injuries and illnesses, disproportionately impacting workers of color and low-wage workers [2]. While it is well established that income, access to healthcare, and health outcomes vary from one community to another, exposures in the workplace are not typically thought of as being differentially distributed in such a way. As a result, studies that have utilized residential neighborhoods, such as the census tract, to examine communities’ social vulnerabilities or environmental exposures typically overlook workplace hazards.

Overlooking workplace hazards can be problematic as it can lead to the assumption that the drivers of health and health disparities in a given community are all happening locally, that is, in the neighborhood of residence. However, for many hazards, such as COVID-19, exposures/transmission may be occurring outside of the residential neighborhood as people engage in different activities throughout the day [3, 4]. Ignoring workplace exposures that occur beyond this local residential context likely misclassifies community exposure by under-counting risks or by obscuring risks entirely.

Racial/ethnic minorities may bear the brunt of this potential exposure misclassification. The COVID-19 pandemic placed a spotlight on how Black, immigrant, and Indigenous communities are disproportionately burdened by workplace exposures, reflecting in part the overrepresentation of these individuals in “essential” workplaces, manual labor, and other low-wage jobs [5, 6]. Workers in these marginalized communities experienced disproportionate risks from COVID-19 in terms of high rates of infection and death [7, 8]. Overlooking how marginalized individuals with correlated exposures in the workplace may cluster in the same residential location likely under-counted the risk of COVID-19 transmission among these communities and probably under-counts other health disparities in the US today. This oversight ensures meaningful social indicators of the workplace, such as hazards faced at work, are not incorporated in place-based indices like the social vulnerability index (SVI) [9], despite work being a social determinant of health and contributing to individuals’ vulnerabilities.

SVI was originally used to identify communities that are vulnerable to poor outcomes following natural disasters and has subsequently been applied to a wide array of health outcomes, including infectious disease outbreaks, with a focus on explaining geographic disparities in disease outcomes [10]. Like many place-based indices, SVI only incorporates information about the immediate local context of a community, such as its socio-demographics, housing stock, accessibility via transit, and other facets of a neighborhood. When workplace exposures are clustered among individuals living in the same location, exposure misclassification may occur in which the impact of workplace exposures that drive geographically clustered risk are misattributed to the attributes of the local community rather than the correlated workplace exposures. Thus, it is pertinent to characterize vulnerable communities beyond the lens of residence-only measures, and to investigate how much additional insight that new indicators of workplace exposures can provide when assessing community health and vulnerability.

In this study, we address this gap by developing place-level measures of occupational hazards. This will allow various stakeholders (e.g., community residents, policymakers) to assess occupational hazards for a particular community or state, which could inform shifts in resources to the most impacted and/or underserved communities that can help set the basis for policies to protect residents. Further, these measures can add to existing indices, such as the SVI or the Environmental Justice Index, which we hypothesize can expand assessments of a community’s vulnerability beyond the immediate local context in a meaningful way. The development of these new occupational metrics informed four primary goals of this study. First, we characterized the distribution of the occupational hazards across the US and whether these hazards cluster among certain communities. Second, we examined whether occupational hazards are systematically under-counted in the SVI. Third, we investigated whether racial/ethnic minorities and low-income individuals are most burdened by occupational hazards. Finally, we examined three markers of health (diabetes, asthma, and high blood pressure) traditionally characterized by disparate burdens on racial/ethnic minority communities [11–15], and whether stratification by exposure to noise, chemical pollutants, and disease/infections, respectively, worsened the observed racial/ethnic health disparity, independent of the SVI, to understand whether these occupational indicators add meaningful information about a community beyond residence-only measures.

## Methods

**Fig. 1:**
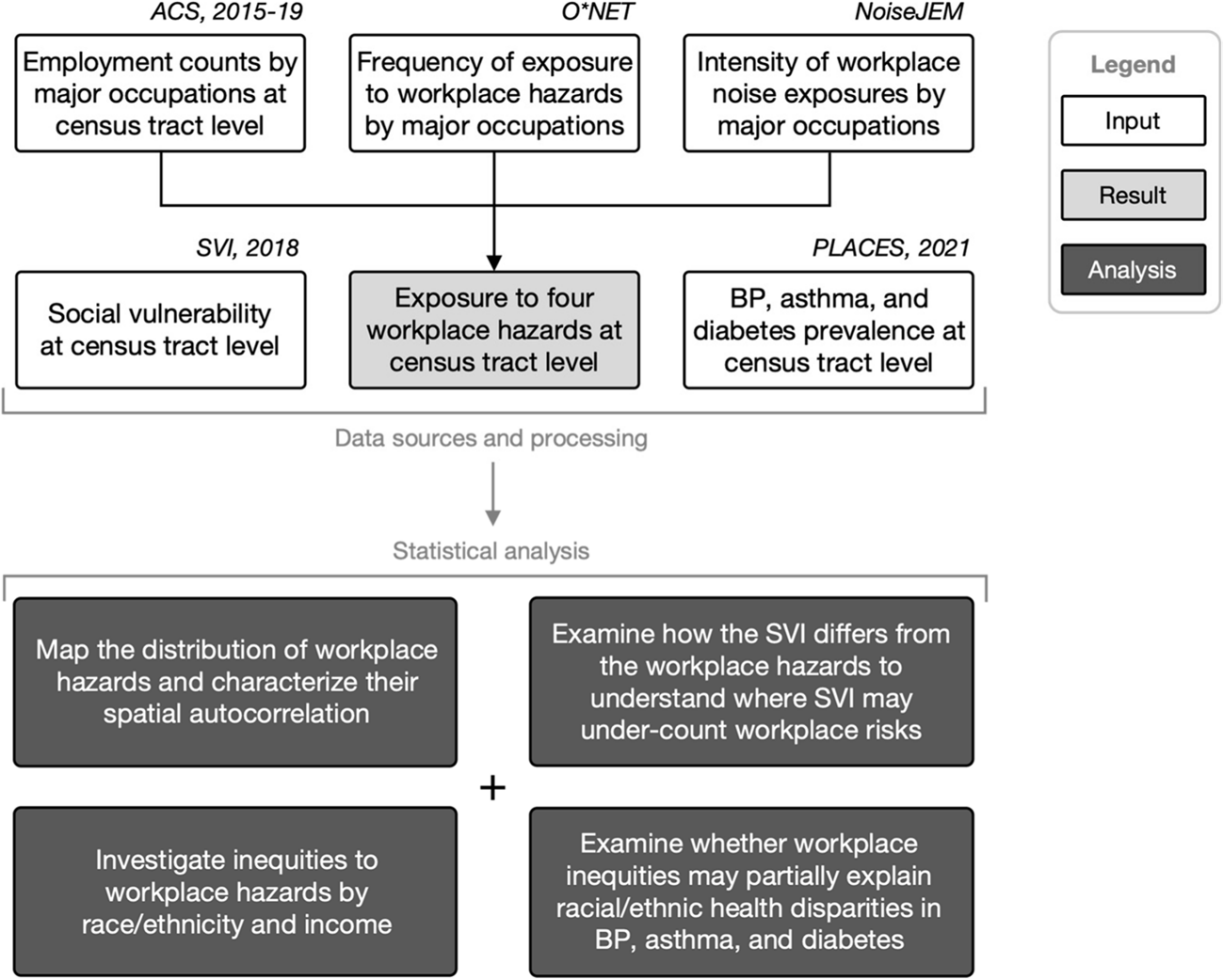
Diagram summarizing the data sources, processing, and statistical analysis. Note: ACS, American Community Survey; O*NET, Occupational Information Network; NoiseJEM, Noise Job Exposure Matrix; SVI, Social Vulnerability Index

We constructed six census tract level occupational hazard indicators covering 72,208 U.S. census tracts. To do this, we used several data sources, including the US American Community Survey (census.gov/programs-surveys/acs), the Department of Labor Occupational Information Network (O*NET; onetcenter.org), and a noise job exposure matrix (JEM; noisejem.sph.umich.edu). All data used in this study were de-identified, publicly available, secondary data. A high-level overview of the methods is illustrated in Fig. 1.

### Creating the Occupational Hazard Indicators

Of the six occupational hazard indicators constructed, five of the indicators were frequency indicators (i.e., the average number of days spent exposed over a given year). The sixth indicator was the estimated percentage of workers exposed to high noise over a given year, which utilized both *frequency* and *intensity* information. To create the six occupational hazard indicators, we leveraged three existing data resources. A brief summary of these resources is provided below.

1. **US Census American Community Survey (ACS)** – we utilized 5-year ACS from 2015 to 2019 to gather total employment count for each US census tract and breakdowns by major occupational groups for 2017. There are 22 major non-military groups. The ACS follows the Census Occupation Code structure for classifying occupational groups and can be linked to the Bureau of Labor Statistics (BLS) Standard Occupational Classification (SOC) structure. The data was collected using the “tidycensus” R package [16].
2. Department of Labor (DoL) O*NET – we accessed DoL O*NET Physical Work Conditions data on the frequency of chemical, physical, and biological hazards for each major occupation code. Chemical hazards were assessed through exposure to pollutants (“How often does this job require working exposed to contaminants (such as pollutants, gases, dust or odors)?”). Physical hazards were assessed through exposure to hazardous equipment (“How often does this job require exposure to hazardous equipment?”), hazardous conditions (“How often does this job require exposure to hazardous conditions?”), and noise exposure (“How often does this job require working exposed to sounds and noise levels that are distracting or uncomfortable?”). Biological hazards were assessed through exposure to disease/infections (“How often does this job require exposure to disease/infections?”) and physical proximity to other workers (a combination of “To what extent does this job require the worker to perform job tasks in close physical proximity to other people?” and “How often do you have to have face-to-face discussions with individuals or teams in this job?”), which, along with exposure to noise, are risk factors for COVID-19 and other infectious disease transmission in the workplace [17, 18]. Each frequency is presented on a continuous scale of 0–100, referred to herein as “Context” scores. The scale is broken down into the following descriptors: 0 is “Never,” 25 is “Once a year or more but not every month,” 50 is “Once a month or more but not every week,” 75 is “Once a week or more but not every day,” and 100 is “Every day.” The data followed the O*NET-SOC structure, which can be cross-walked with the BLS SOC structure.
3. **NoiseJEM** – we used the US/Canada noise job exposure matrix (NoiseJEM) to estimate workplace noise intensity by job category. The JEM contains mean, median, minimum, maximum, and standard deviation 8-h time-weighted averages (TWA-8 h) levels measured according to the criteria of the National Institute for Occupational Safety and Health (NIOSH) and Occupational Safety and Health Administration (OSHA) from 1963 to 2015. Exposure estimates were available across all jobs and industries within the JEM. Classification of job titles followed the BLS SOC structure. Measurements using OSHA Permissible Exposure Limit samples were used for analysis in this study.

To estimate occupational hazards at the census tract level, we first estimated the count of workers in each of the major census occupational groups within each census tract. Five-year, 2015–2019 ACS estimates were used to be consistent with the years of data available in the other data sources. Next, we estimated the frequency of exposure to chemical pollutants, disease/infections, hazardous equipment, noise exposure, general hazardous conditions, and physical proximity to other people using O*NET. The construction of these frequency indicators is detailed in Appendix A of the Supplemental Materials. Briefly, a weighted score for each census tract is created by multiplying the proportion of workers by major occupational groups with the average frequency of exposure for each hazard and summed by census tract. These scores can be interpreted as the number of days a typical worker in this census tract is exposed to a given occupational hazard. By dividing the number of days by the 250-day standard work-year, this score could also be interpreted as the percentage of days in a standard work-year the typical worker is exposed.

Appropriately characterizing exposures requires accounting for their frequency, intensity, and duration. Since O*NET assesses only the frequency of exposure to hazardous noise at work, we employed data from NoiseJEM to estimate the intensity and duration of this exposure at the census tract level. The process employed to construct this index is similar to a Monte Carlo simulation approach described in a previous paper [19], expanding the methodology to the census tract level. Appendix A of the Supplemental Material provides additional detail on the construction of our occupational indicator as well as the Monte Carlo simulation approach. This indicator can be interpreted as the percentage of workers in a given census tract exposed to hazardous levels of noise at work.

Overall, we created six occupational hazard indicators for each census tract in the US. Specifically, these measure the average number of days exposed to (1) chemical pollutants, (2) hazardous equipment, (3) hazardous conditions, (4) infectious disease/infections, and (5) physical proximity with other workers, and (6) the prevalence of hazardous workplace noise exposure.

### SVI and Health Metrics

Census tract level estimates of the 2018 SVI were used to evaluate the relationship between the occupational indicators and SVI. The SVI score is presented as a percentile, where a higher percentile indicates higher social vulnerability of socioeconomic status (e.g., percent unemployed), household characteristics (e.g., aged 65 and older), racial/ethnic minority status (e.g., percent Hispanic or Latino), and housing type and transportation (e.g., percent of mobile homes). Census tract level metrics of health from the CDC PLACES Project 2021 data release [20] were used to examine whether our estimated occupational hazard indicators were associated with key health status indicators across the US. Specifically, we extracted census tract level prevalence’s of high blood pressure, asthma, and diabetes.

### Statistical Analysis

All analyses were conducted using R v4.0.2 [21] and Geoda [22]. Census tract level maps were constructed using the “tigris” and “sf” R packages [23, 24]. Summary statistics (medians, interquartile ranges (IQRs), percentiles) were used to describe the data. We first characterized the distribution of the occupational hazards across the US and whether these hazards cluster among certain communities in the US. Pearson’s correlations were used to assess associations between occupational indicators to examine whether any indicators were redundant. Global univariate Moran’s *I* was calculated to assess the spatial autocorrelation of occupational indicators nationwide and for each US state using the “spdep” R package [25]. Univariate local indicators of spatial autocorrelation (LISA) were calculated to understand where concentrated clusters of the occupational indicators using the “rgeoda” R package [26]. Global bivariate Moran’s *I* was calculated between each of the occupational indicators to examine how each occupational indicator was correlated with its neighbor of another occupational indicator. Moran’s *I* follows a similar range to traditional correlation (− 1 to 1), where 1 suggests high, positive spatial autocorrelation.

Next, to understand how the SVI differs from occupational exposures, we visualized the difference in the average percentile of the occupational indicators and the SVI score. Bivariate LISA was conducted using the occupational indicators and the SVI score to further examine concentrated clusters of high occupational scores, but low SVI scores in the US. This high exposure–low SVI group demonstrates if and where the SVI may overlook/under-count occupational exposures.

We then examined relationships between the occupational indicators with tract level distributions of race/ethnicity, income, and the average percentage of racial/ethnic minorities and low-income individuals. This will allow us to better understand whether occupational exposures overburden marginalized communities in the US. This was done by investigating the percentage of the “worst-off,” or most highly occupationally exposed (> = 95th percentile), census tracts by various categories of race/ethnicity and income. We used simple, unadjusted natural cubic splines with four degrees of freedom to further understand the relationship between these three socio-demographics with each occupational indicator. No random effects or spatial weights matrix was included in the models to account for residual spatial autocorrelation; while ignoring spatial autocorrelation may shrink regression models’ standard errors toward the null, the intention of the spline models was to capture general associations rather than examining the statistical significance of our analyses.

Pearson’s correlation was used to assess associations between occupational indicators and prevalence of various health outcomes to validate that occupational exposures are correlated with adverse health, as would be expected. We then examined whether place-based occupational indicators add meaningful information about a community beyond residence-only measures by investigating racial/ethnic disparities in three health outcomes (diabetes, asthma, and high blood pressure). The prevalence of these three health outcomes was modeled using Poisson regression and a natural cubic spline with four degrees of freedom for the percentage of racial/ethnic minorities. Each relationship was stratified by high/low noise exposure, chemical pollutant exposure, and disease/infection exposure, respectively, by splitting census tracts by > 50th percentile (high) vs < 50th percentile (low) of exposure. We ran two model types: (1) adjusted by SVI score to understand whether between-place variation in the outcomes is captured by the occupational risk metrics independent of local variation in SVI, and (2) unadjusted by SVI to examine the crude relationship between occupational exposures with the outcomes. Similar to the other splined models, no random effects or spatial weights were accounted for in the models. These exposure-health outcome pairs were selected due to the well-characterized racial/ethnic disparities in diabetes [11, 12], asthma [13], and high blood pressure [14, 15], as well as their established exposure–response relationships [27–31].

## Results

### Overview of Occupational Indicators

**Fig. 2:**
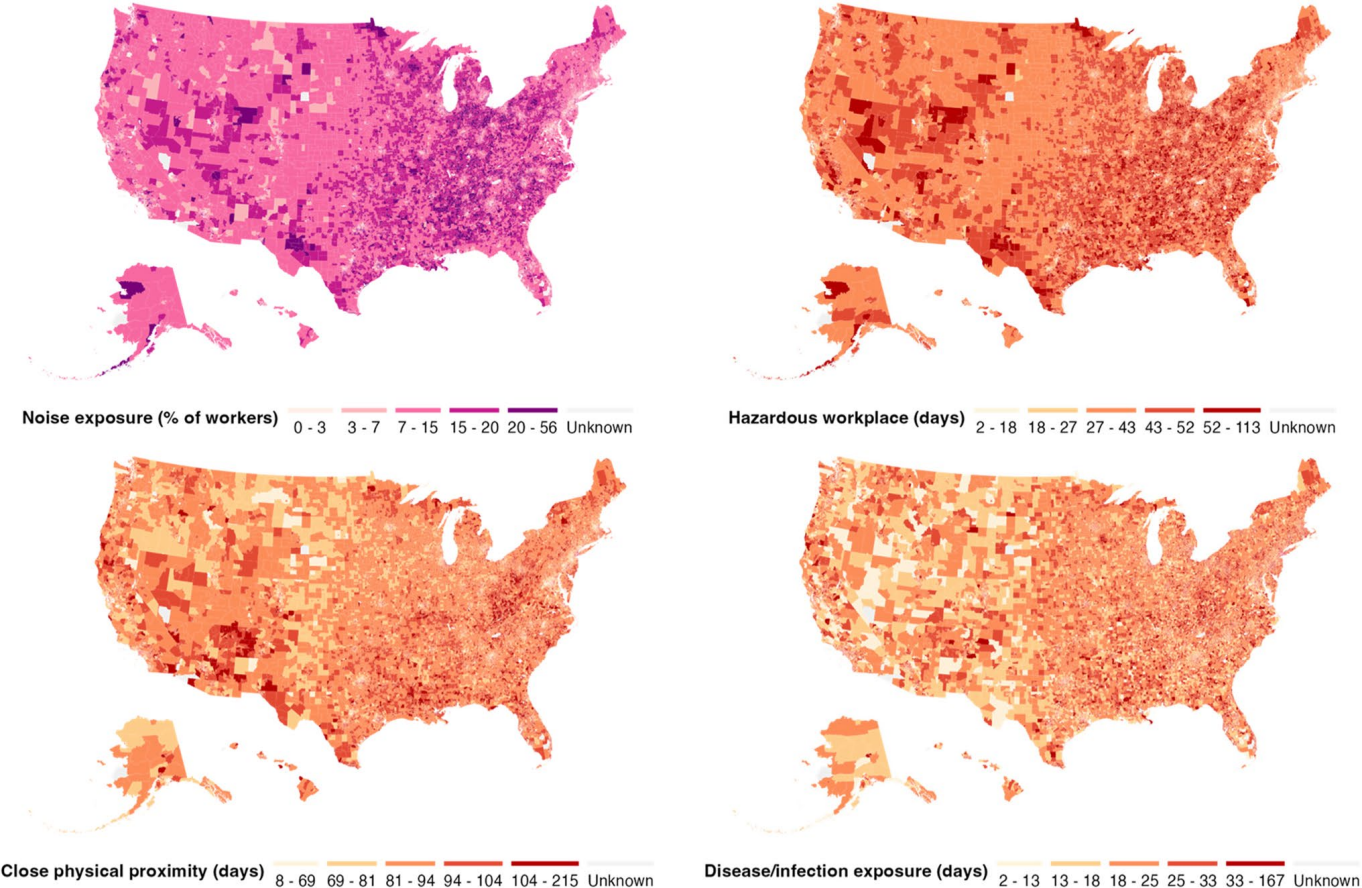
Prevalence of work-related noise exposure, average days in hazardous workplaces, average days in close physical proximity with other workers, and average days exposed to disease/infection across 72,208 census tracts in the US with working population > 20 workers. The maps display each indicator’s 0–10th percentile, 10–25th percentile, 25–75th percentile, 75–90th percentile, and 90–99th percentile

We estimated census tract level indicators of occupational hazards for 72,208 US census tracts. Descriptive statistics for each occupational hazard indicator are displayed in Fig. 2 and detailed in Supplemental Table S1. Over the course of a standard 250-day work-year, the median worker in the US spent 88 days (IQR = 81–94) in close physical proximity to other workers, equivalent to 35% (IQR = 32–38%) of the year. We estimated that the median worker was exposed to chemical contaminants for 22% (IQR = 17–26%) of the year, hazardous equipment for 12% (IQR = 9–15%) of the year, and hazardous conditions for 8% (IQR = 7–10%) of the year. The median worker in the US spent the fewest days at risk of disease/infection exposure at work (8%, IQR = 7–10%). The median prevalence of hazardous noise exposure among employed residents in US census tracts was 11% (IQR = 7–15%).

Each of the six occupational indicators displayed moderate univariate spatial autocorrelation (0.30 ≤ *I* ≤ 0.66) (Supplemental Table S2), indicating that communities with high occupational exposures tend to cluster together. Among the occupational hazard indicators, exposure to noise, chemical contaminants, hazardous equipment, and hazardous conditions were all positively correlated, with all pairwise relationships correlated at a level above *r* = 0.95 (Supplemental Table 2). Similar to Pearson’s correlation, exposure to noise, chemical contaminants, hazardous equipment, and hazardous conditions all indicated positive, bivariate spatial autocorrelation, with all pairwise relationships at a level above *I* = 0.63, indicating that communities exposed to multiple occupational exposures tend to cluster together. This informed the creation of an aggregated occupational indicator of the chemical pollutants, hazardous equipment, and hazardous conditions indicators, referred to herein as the hazardous workplace indicator, by taking the average of these three indicators.

Physical proximity with other workers was moderately correlated with other occupational indicators (0.45 ≤ *r* ≤ 0.54) and had moderate bivariate spatial autocorrelation with them (0.30 ≤ *I* ≤ 0.46). On the other hand, exposure to disease/infections was weakly and negatively correlated most other occupational indicators chemical contaminants, indicators (− 0.20 ≤ *r* ≤ 0.45), and similarly weak, but positive, for spatial autocorrelation (0.03 ≤ *I* ≤ 0.30) (Supplemental Table S2).

States in the Midwest and West displayed the strongest spatial autocorrelation (0.6 ≤ *I* ≤ 0.8) for exposure to noise and the average hazardous workplace indicator (Supplemental Fig. S1). Physical proximity to other workers displayed moderate spatial autocorrelation (0.4 < *I* < 0.6) in Northeast and Southern states, and was observed highest in Washington, DC (*I* = 0.61). Exposure to disease/infection at work was the least spatially autocorrelated occupational indicator statewide, although moderate spatial autocorrelation was observed in New York and Minnesota. Local indicators of spatial autocorrelation are shown in Supplemental Fig. S2.

### Relationship Between SVI and Occupational Exposure

**Fig. 3:**
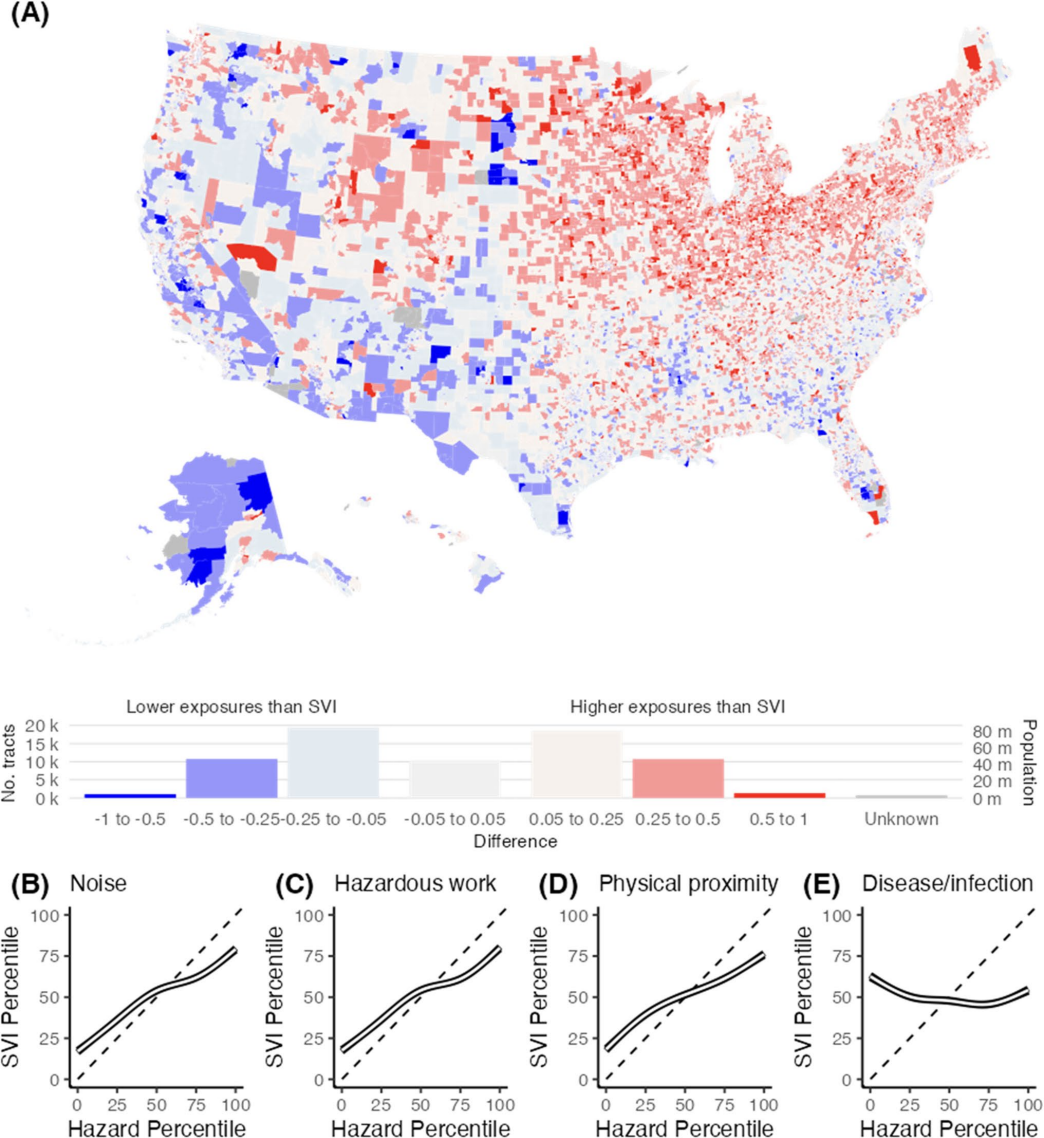
**A** Difference in average nationwide percentile of occupational indicators and the nationwide social vulnerability index (SVI) percentile; values above 0 indicate a higher percentile occupational exposure than the SVI percentile. **B**–**E** Unadjusted association between four occupational indicators and the SVI modeled using a natural cubic spline with four degrees of freedom of (**B**) work-related noise exposure, **C** average days in hazardous workplaces, **D** average days in close physical proximity with other workers, and (**E**) average days exposed to disease/infection

Figure 3A displays how the average percentile of the four occupational indicators relates to the SVI score by taking the differences between the two. There were 12,026 census tracts (16.5%) that had a difference in occupational exposures compared to their SVI score by at least 0.25 (Fig. 3A and Supplemental Table S3), indicating nearly 1 in 6 census tracts may be substantially under-counting occupational risks. On average, census tracts in the Midwest had higher occupational exposure percentiles than their SVI score (mean = 0.09, SD = 0.25), while tracts in the West had a slightly lower difference (mean =  − 0.09, SD = 0.23). Tracts in the Northeast and South had non-substantial differences. Rural tracts (with population density < 1000 people/m^2^) had a slightly higher difference (mean = 0.11, SD = 0.24), while dense, urban tracts (with population density > 10,000 people/m^2^) had a slightly lower difference on average (mean =  − 0.18, SD =  − 0.20).

Specifically examining the bivariate LISA of each of the four occupational indicators with the SVI to identify areas with high workplace exposure surrounded by areas with low SVI, reflecting areas that may be under-counting risks (Supplemental Fig. S3), there were 2731 (3.7%) census tracts that had high workplace noise exposure and low SVI, disproportionately concentrated in the Midwest. Similar trends were observed for the aggregated hazardous workplace indicator and the close physical proximity indicator. The trends were more pronounced when examining exposure to disease/infection. There were 7790 (10.6%) census tracts with high disease/infection exposure and low SVI, disproportionately concentrated in the Northeast and Midwest.

Figure 3B–E shows relationships between the percentiles of four occupational indicators with SVI percentiles using natural cubic splines. The dashed lines show how occupational exposure and SVI would increase together if there was perfect, 1-to-1 correspondence between them. Generally, an increase in the percentage of workers exposed to hazardous noise, the percent of work-year in hazardous workplaces, or the percent of the work-year spent in close physical proximity was associated with an increase in SVI. However, census tracts with high exposures to these three occupational hazards (> 75th percentile) coincided on average with lower-than-average SVI percentile (50–75th percentile), and vice versa for census tracts with low exposures (< 25th percentile). This discrepancy was even more profound when examining the relationship with exposure to disease/infections.

### Racial/Ethnic and Income Inequities in Occupational Exposures

**Fig. 4:**
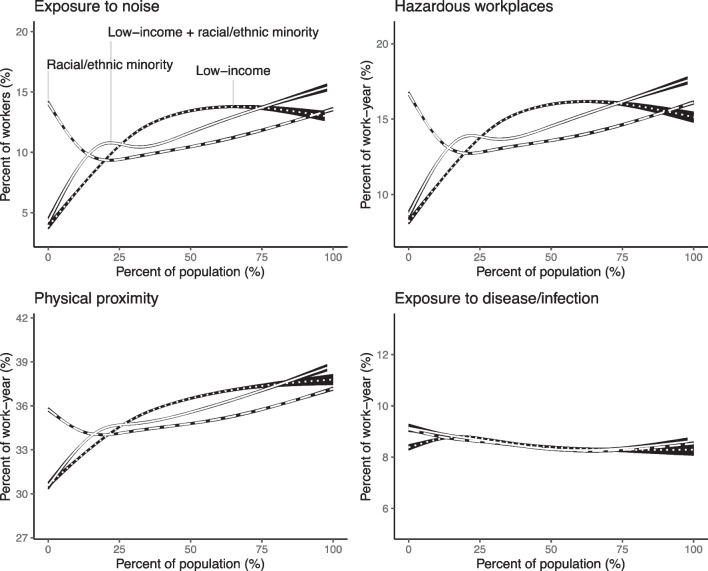
Relationship between percent of racial/ethnic minority, percent of low-income individual, or the average percent of low-income individual and racial/ethnic minority and exposure to four occupational hazards, modeled using a natural cubic spline with four degrees of freedom

We examined how the four main occupational indicators varied by the percent of racial/ethnic minorities, low-income individuals, and the average percentage of the two (Fig. 4). Generally, an increase in the percentage of racial/ethnic minorities in a census tract was associated with an upward U-shaped relationship with exposure to noise, hazardous workplaces, and close physical proximity to other workers. Both census tracts with nearly no racial/ethnic minorities and nearly entirely racial/ethnic minorities were the most highly exposed three occupational exposures. Meanwhile, these three exposures displayed a more strong, sigmoidal relationship with the percent of low-income individuals in a census tract. When considering the average percentage of racial/ethnic minorities and low-income individuals together, these three exposures were associated with a positive, generally linear relationship (Fig. 4A–C), indicating census tracts with a higher proportion of both racial/ethnic minorities and low-income individuals may be the most highly exposed to noise, hazardous workplaces, and close physical proximity to other workers. On the other hand, neither the percentage of racial/ethnic minorities alone, nor the percentage of low-income individuals, displayed an association with exposure to disease/infections (Fig. 4D).

Investigating the “worst-off,” most high-occupationally exposed (> = 95th percentile) census tracts (Supplemental Table S4), between 7.9 and 14.6% of the worst-off census tracts were in communities with > 75% racial/ethnic minorities, while just 2.0–3.1% of the most highly exposed census tracts were in tracts with < 25% racial/ethnic minorities for each of the four occupational indicators. A similar, but wider disparity was observed when considering either the percent of low-income individuals, or the average percentage of racial/ethnic minorities and low-income individuals (Supplemental Table S4).

### Racial/Ethnic Health Disparities in Occupational Exposure

**Fig. 5:**
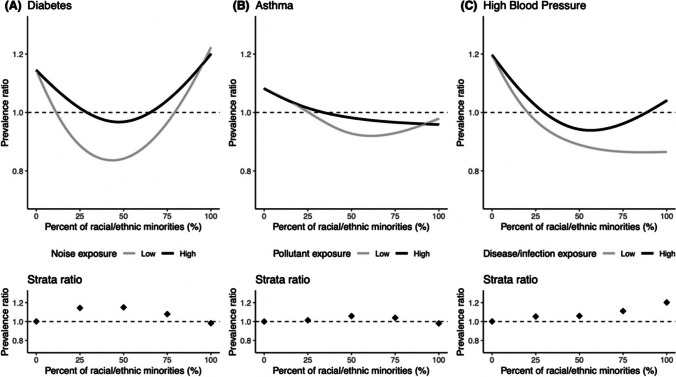
Social Vulnerability Index–adjusted relationship between census tract percentage of racial/ethnic minorities and the prevalence ratio of (**A**) diabetes, **B** asthma, and (**C**) high blood pressure using a natural cubic spline with three degrees of freedom. Each relationship was stratified by high/low noise exposure, chemical pollutant exposure, and disease/infection exposure, respectively, by splitting census tracts by > 50th percentile (high) vs < 50th percentile (low) of exposure. Bottom plots show the prevalence ratio within strata of high vs low exposure tracts

Pairwise correlations between the occupational indicators and high blood pressure, asthma, and diabetes all showed positive associations, albeit weakest for exposure to disease/infections (Supplemental Table S5). The splined relationship between the percentage of racial/ethnic minorities and the prevalence ratio of these three health markers, stratified by high/low exposure to the respective occupational indicators, generally shows a higher percentage of racial/ethnic minorities is associated with a more strongly positive, increased prevalence of diabetes, asthma, and high blood pressure among highly exposed tracts compared to less-exposed tracts (Supplemental Fig. S4). After adjusting for the SVI, the modeling demonstrated that health disparities persist between low- and high-occupationally exposed census tracts (Fig. 5). Specifically, high-occupationally noise-exposed tracts had higher prevalence of diabetes than low-occupationally noise-exposed tracts among tracts with 50% racial/ethnic minorities. A similar, attenuated disparity was observed on the prevalence of asthma as a result of chemical pollutant exposure at work, as well as the prevalence of high blood pressure as a result of disease/infection exposure at work.

## Discussion

Exposures to workplace hazards are often overlooked in geospatial analyses of health focused on residential location as a driver of contextual risk. In this study, we developed an approach for the nationwide estimation of workplace hazard exposures indexed to residential location and observed how current assessments of social vulnerability may overlook communities with high workplace exposures. The implication of overlooking these communities was highlighted by examining how racial/ethnic health disparities in diabetes, asthma, and hypertension may be partly explained by inequities in workplace exposures, capturing the between-place variation of health outcomes beyond what the SVI already captures. Collectively, the findings suggest that the current approach of only incorporating residential location indicators of risk may systematically under-count risks when attempting to identify the most vulnerable communities in the US in measures like the SVI.

Many occupational exposures (e.g., workplace noise, contaminants, hazardous equipment, hazardous conditions, and close physical proximity) are spatially concentrated according to our study findings. Communities with similar exposure to workplace hazards are generally situated near one another, supporting the notion that industrialization, which exposes workers to various chemical, physical, and safety hazards, tends to cluster marginalized communities together and supporting the notion that occupational exposures should also be viewed with a place-based lens. Previous evidence for this is very limited. A case study of occupational groups in Bronx, NY, provides cursory support for this clustering of occupational exposures; white color jobs were found to generally cluster in northeast Bronx, while service and manufacturing jobs tended to cluster in southwest Bronx [32]. This study extends these findings to a nationwide scale and investigating exposures and hazards beyond just examining employment of occupational groups.

To investigate whether some occupational indicators are redundant, we examined correlations between each indicator. Tract-level exposure to contaminants, hazardous equipment, noise, and hazardous conditions were all strongly correlated with one another, while the physical proximity and disease/infections indicators generally were less so. The strong correlations between these four measures may indicate that workers in high hazard industries are often exposed to multiple hazards simultaneously [33, 34]. While the combination of these four occupational indicators may result in over-counting of occupational exposures, inclusion of two or three of these highly correlated occupational indicators in indices such as the SVI may be appropriate, despite the correlation, as it reflects the reality of the multiple hazards workers to which workers may be exposed. For the purposes of our study, we averaged three of the indicators into one, “hazardous workplace” indicator. Future analyses should consider using either exposure to hazardous conditions or hazardous equipment use, since both indicators are metrics of general workplace safety and injury.

The findings showed that there were also thousands of census tracts across the US with high workplace exposures (particularly exposure to disease/infections) yet were adjacent to tracts with low SVI. This suggests that not accounting for workplace hazards within risk indices such as the SVI may under-count community risks when trying to identify the most vulnerable neighborhoods, particularly in future disease outbreaks like COVID-19 where the workplace may once again be a critical indicator of community risk [35]. Incorporating indicators of workplace hazards into these risk indices provides a different lens to examining a community’s vulnerability beyond the risks within the immediate local context, as workplace hazards are often experienced far away from an individual’s residence or community. This was evidenced by the health disparities observed between low- and high-occupationally exposed communities, persisting even after accounting for SVI score. The occupational indicators constructed in this study may be used to better understand the between-place variation in health outcomes beyond what currently available indicators can currently capture. However, the extent varied across occupational risks and future research is required to understand the true extent of this between-place variation.

Comparing occupational scores to the prevalence of various adverse health outcomes indicated moderate associations for most occupational indicators. Exposure to disease/infections and close physical proximity to other workers were weakly associated with the non-communicable disease outcomes in this study; however, both of these indicators may be more relevant for examining spatial disparities in other health outcomes such as COVID-19 [35]. Regardless, the findings suggest the indicators may be valid measures of occupational hazards and they provided some evidence of how workplace exposures may result in health disparities. Our study showed how racial/ethnic minority communities disproportionately work in more hazardous jobs. Furthermore, when considering the intersection of low-income alongside racial/ethnic minorities, the disparities widened, as has been shown in other environmental health studies [36]. This exposure inequity likely leads to health disparities; this was further evidenced in our study by demonstrating how even among majority racial/ethnic minority census tracts, those with high levels of occupational exposure exhibited greater prevalence of diabetes, asthma, and hypertension than less-exposed tracts.

Our study’s observation of sociodemographic occupational inequities emphasizes how the workplace contributes to racism as a fundamental cause of health inequalities in the US [37]. Link and Phelan defined fundamental cause as “resources that determine the extent to which people are able to avoid risks for morbidity and mortality” [38]; in the workplace, racial/ethnic minorities are disproportionately exposed to workplace hazards which are unavoidable. Since racial/ethnic minorities continue to remain occupationally segregated in jobs with lower power and prestige [39], they also typically have less resources in the workplace to avoid workplace hazards through engineering controls and personal protective equipment. This was highlighted during the COVID-19 pandemic among healthcare workers, where practitioners had the resources to adequately protect themselves from infection, while lower-wage, healthcare support workers did not [40–42], despite both occupational groups having similar risks of infection and transmission. This may help explain why the disease/infection indicator did not display strong associations with either racial/ethnic minorities or SVI; practitioners such as physicians or nurses with considerable infection risk are likely to have lower social vulnerability and may dilute the observed associations toward the null. These inequities in workplace resources have affected workers long before the COVID-19 pandemic and will continue to impact workers in the future. Thus, workplace hazards are an important, yet commonly overlooked, factor of social vulnerability that must be investigated to reduce health disparities in the US.

Our study has some limitations that are important to consider when interpreting our results. The five frequency indicators constructed for this study are a weighted average over all employees in a given tract. While the weighting allows for a more representative estimation, our method did not incorporate variability in our estimates. Developing a method that incorporates variability would allow for more confidence in the results, although there would still be no simple and efficient way to validate them. Furthermore, due to limitations in the granularity of employment count at the census tract level, weighted frequency estimates were constructed using major occupational groups. There could be substantial variability in workplace exposure and availability of workplace controls/personal protective equipment at more granular occupational grouping levels, such as at the minor or detailed level (e.g., management as a major group vs top executives as a minor group), that can influence adverse health outcomes, as shown from health inequalities during COVID-19 [43]. In the future, we plan to address this limitation by using estimates of employment count of minor or broad occupational groups at a broader spatial level, such as a metropolitan/nonmetropolitan area or state, to weight the construction of average frequency exposure by minor/detailed occupational groups, as well as by including margins of error in the original estimation of employment at the tract level.

Low employment count in some tracts makes it difficult to estimate a true prevalence of overexposure to hazardous workplace noise. Our estimations also made use of a national JEM that does not incorporate spatial differences in measurements and instead assumes that exposures for the same job are not influenced by place. However, our Monte Carlo approach, combined with the efficient use of public occupational data through JEMs and the O*NET, has allowed us for the first time to estimate overexposure to hazardous workplace noise exposure at the census tract level. This methodology also provides a framework for replicating this strategy for other workplace exposures of particular concern, such as asbestos, silica, and various carcinogenic and non-carcinogenic chemicals, using other JEMs, and highlights the importance of developing JEMs for researcher and practitioner use. Such prevalence estimates of overexposure to various chemicals could also be easily updated over time as more data is fed into the JEMs, and would allow for temporal trends in overexposure prevalence to be evaluated once sufficient data are available over time. We recommend that future JEMs feature a column related to the place the measurement was collected at the lowest level reasonably possible (i.e., if census tract may raise concerns around privacy, collection might occur at the county or state level). This would allow a more accurate estimation of overexposure prevalence across place. The greatest strength of our Monte Carlo approach to estimating overexposure prevalence is that it allowed for better estimation of complete exposures, that is, it incorporates the frequency, intensity, and duration of workplace exposures in its estimates and incorporated variability in the estimation. Furthermore, since the estimates created a distribution of exposures for every worker, this method allows for simple aggregation of the estimates to larger spatial levels, such as county or state, by aggregating the distributions. Future analysis could increase the number of Monte Carlo iterations to improve the precision of the estimates, although our current use of 100 iterations produced relatively precise estimates.

This strategy is still susceptible to the modifiable areal unit problem. While these estimates are not point-based measures, the construction of these measures is based on aggregated estimates of employment count for an arbitrary administrative boundary—the census tract. However, the construction of these estimates is tied fundamentally to employment count estimates at the tract level, which is not tied to a specific point in space within a census tract (e.g., the address of one’s home), but rather is meant to reflect the individual living in their respective arbitrary census tract. Further, the integral use of employment counts allows for estimation of each indicator at multiple, arbitrary spatial levels, as employment count at the county-, metropolitan-, state-, and US-level can be utilized in place of tract level estimates.

Together, these findings show that development of occupational indicators at different spatial levels is feasible, that disparities in occupational exposures may explain health disparities in the US, and that indicators of occupational hazards could be incorporated in geospatial analyses and place-based indices such as the EPA EJScreen and the Social Vulnerability Index. Otherwise, we may be under-counting communities’ health risks and social vulnerability to disease.

## Supplementary Information

Below is the link to the electronic supplementary material.

Supplementary file1 (DOCX 30425 KB)

## Data Availability

Publicly available data was used for this work: (1) American Community Survey, https://www.census.gov/programssurveys/acs; (2) Department of Labor Occupational Information Network, https://www.onetcenter.org/; (3) NoiseJEM, https://noisejem.sph.umich.edu/; (4) Center for Disease Control Social Vulnerability Index, https://www.atsdr.cdc.gov/placeandhealth/svi/data_documentation_download.html; and (5) Center for Disease Control PLACES Project, https://www.cdc.gov/places/index.html.
