# Supplementary material for: Workplace Exposures Vary Across Neighborhoods in the US: Implications on Social Vulnerability and Racial/Ethnic Health Disparities
Source: J Racial Ethn Health Disparities. 2024 Aug 30;12(5):3388–400. doi: 10.1007/s40615-024-02143-5 (PMC12446402; doi:10.1007/s40615-024-02143-5)
Supplement: Supplementary file 1 — Supplementary file1 (DOCX 30425 KB) [file 40615_2024_2143_MOESM1_ESM.docx]

# Supplementary Material for

# Title: Workplace exposures vary across neighborhoods in the US: implications on social vulnerability and racial/ethnic health disparities

# Abas Shkembi, MS^1,*^; Jon Zelner, PhD^2,3^; Sung Kyun Park, ScD^1,2^; Richard Neitzel, PhD, CIH, FAIHA^1^

# ^1^Department of Environmental Health Sciences, University of Michigan School of Public Health, Ann Arbor, MI, USA

# ^2^Department of Epidemiology, University of Michigan School of Public Health, Ann Arbor, MI, USA

# ^3^Center for Social Epidemiology and Population Health, University of Michigan School of Public Health, Ann Arbor, MI, USA

# *Address correspondence to: Abas Shkembi, 1415 Washington Heights, University of Michigan School of Public Health, Ann Arbor, MI 48109 (e: [ashkembi@umich.edu](mailto:ashkembi@umich.edu))

**Supplemental Material**

Appendix A. Construction of the occupational indicators

Context scores from O*NET are difficult to interpret; further, a one-unit change in a context score is not the same as a one-unit change the days within a year, which is what context scores are measuring. To make this measure easier to interpret, we translated context scores into day-equivalents (for a typical 250 day work-year) using the relationship in **Figure A1**.

| 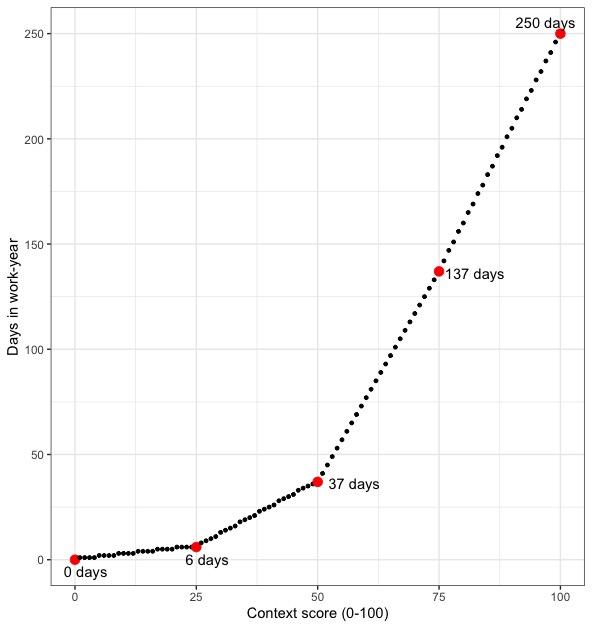 |
| --- |
| **Figure A1.** Constructed relationship between context scores and days exposed in a standard work year (250 days). O*NET scores range from 0 – 100 (0: never; 25: Once a year or more but not more than every month; 50: Once a month or more but not every week; 75: Once a week or more but not every day; and 100: Every day). Assuming a 250 day, standard work-year, context scores were reassigned to equivalent work days (Context score of 0: 0 work days; Context score of 25: midpoint of 1-12 work days, or 6 days; Context score of 50: midpoint of 12-50 work days, or 31 days, plus the 6 previous work days for a total of 37 days; Context score of 75: midpoint of 50 and 150 work days, or 100 days, plus the previous 37 work days for a total of 137 days; and finally, Context score of 100: 250 work days). |

O*NET provides data at the detailed occupation level, so we estimated average days exposed to a particular hazard for each major occupational group and referred to this as the mean frequency score, *F_i_*, for a given major occupation group *i*. This information was then joined with employment count estimates of the major occupation groups by census tract. The employment counts were converted to proportions within a census tract, and a *weighted* frequency score for each major occupation group within a census tract was created by multiplying the proportion of those employed by the mean frequency scores. These weighted frequency scores were then summed within a given census tract to assign a single, summed weighted frequency score to that tract for each of the occupational hazards. The construction of the weighted frequency score for the frequency of an occupational exposure, *O*, for a given census tract, *i*, can be described by Equation 1 below:

$O_{i}= \sum_{j=1}^{22} p_{ij}\cdot F_{j}$ (1)

where *p_ij_* denotes the proportion of workers belonging to major occupation group *j* in census tract *i*. *F_j_* denotes the mean frequency score for major occupation group *j* (*j* = 1, 2, 3, …, 22; note there are 22 major non-military occupation groups). For example, imagine a census tract with 50% of its working population in service occupations, and the other 50% in farming, fishing, and forestry. We know from O*NET that the mean frequency scores for exposure to chemical contaminants for these major occupation groups is 20 days and 60 days, respectively. We can then create a summed, weighted frequency score for this tract as follows: (0.5*20) + (0.5*60) = 40 days. This process can then be repeated for each census tract and each occupational hazard class. Specifically for the creation of the physical proximity indicator, *F_j_* was adjusted by multiplying the frequency of face-to-face discussions by an indicator of the physical proximity question (1: >62.5, or halfway between “Slightly close” and “At arm’s length”; 0: ≤62.5).

Next, we created the prevalence of exposure to hazardous noise indicator. **Figure A2** summarizes the overall process. Using posterior distributions of each major occupation group from a hierarchical Bayesian imputation analysis of NoiseJEM,^[[1]](#footnote-1)^ we sampled noise exposures equal to the number of workers within a major occupation group in a given census tract 100 times using a Monte Carlo simulation approach. Using these simulated exposures, we estimated the maximum allowable exposure time in the simulated workplace exposures over a given year under the assumptions that any given individual is exposed to the same noise levels every day, and that they work a typical 2000-hour (i.e., 250 8-h days) work-year. The maximum allowable exposure time can be calculated using the following equation:

$T_{allowed}=\frac{2000}{2^{\frac{(TWA-85)}{5}}}$ (2)

Where exchange rate is 5 dB and an occupational exposure limit of 85 dBA (to which overexposure is associated with increased risk of hearing loss).

| 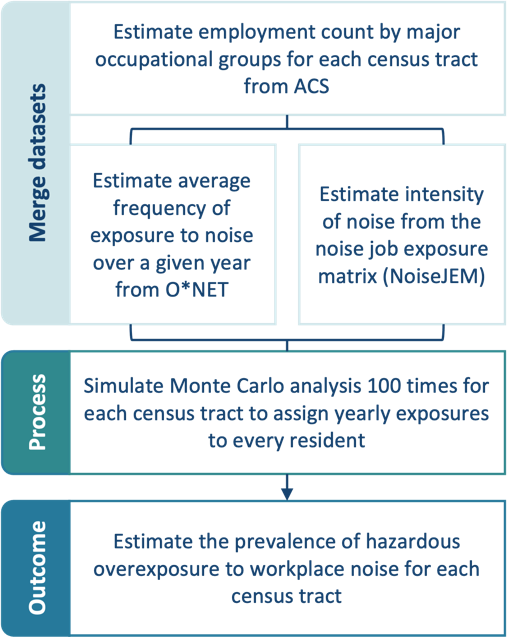 |
| --- |
| **Figure A2.** Monte Carlo workflow to estimate prevalence overexposure to workplace noise by census tract. |

It is worth noting that while OSHA PEL noise measurements were used in this analysis, we used the occupational exposure limit (85 dBA) associated with the OSHA Action Level (AL) to determine overexposure as the OSHA PEL is not sufficiently protective of workers, and is less protective than the OSHA AL. Using the mean frequency score *F_i_* for a given major occupation group, we converted the average days exposed to uncomfortable noise levels to hours (by multiplying days by 8 hours) to estimate the time exposed to hazardous noise levels for each major occupation group. Dose can then be estimated for any worker as a ratio of time exposed (assumed to be a standard 8-hr shift for each individual) to time allowed, such that $Dose=100\times\frac{T_{exposed}}{T_{allowed}}$. From there, we estimated the percent of workers exposed to hazardous levels of noise over a typical work year for each census tract. We considered Dose >50% to be “hazardous” (which would equate to >80 dBA TWA-8h) for two reasons: (1) an industrial hygienist would deem such exposures “unacceptable” in the workplace,^[[2]](#footnote-2)^ and (2) Bayesian analyses of workplace exposure assessments have suggested that any average exposure within 50%-100% of the occupational exposure limit are highly likely to have a true exposure profile of Dose >100%.^[[3]](#footnote-3)^ Using the 100 Monte Carlo simulations, we calculated the mean percent of workers exposed to hazardous occupational noise and used this mean in subsequent analyses. Hearing protection device (HPD) use was not incorporated in the Monte Carlo simulation as the extent of workplace exposures should be examined prior to considering any control (e.g., PPE, ventilation) to understand the full scope of the problem.

Appendix B. Supplemental Tables and Figures

| **Supplemental Table S1.** Descriptive statistics of occupational exposures across 72,208 census tracts in the US with working population > 20 workers. | | | |
| --- | --- | --- | --- |
|  | **Median** | **IQR** | **Min-Max** |
| **Frequency (of 250 standard work-days)** |  |  |  |
| Physical proximity (in days) | 88 | 81-94 | 8-215 |
| *% of standard 250 days* | *35%* | *32-38%* | *3-86%* |
| Chemical pollutants | 54 | 43-65 | 3-137 |
| *% of standard 250 days* | *22%* | *17-26%* | *1-55%* |
| Hazardous equipment | 30 | 22-38 | 1-108 |
| *% of standard 250 days* | *12%* | *9-15%* | *<0.5-43%* |
| Hazardous conditions | 21 | 17-25 | 1-69 |
| *% of standard 250 days* | *8%* | *7-10%* | *<0.5-28%* |
| Disease/infections | 21 | 18-25 | 2-169 |
| *% of standard 250 days* | *8%* | *7-10%* | *1-67%* |
|  |  |  |  |
| **Prevalence (% of working population)** |  |  |  |
| Hazardous noise (>80 dBA) | 11% | 7-15% | 0-56% |

| **Supplemental Table S2.** Bivariate correlations (Pearson’s *r*), univariate spatial autocorrelation (Moran’s *I*) and bivariate spatial autocorrelation (Moran’s *I*) among six occupational indicators | | | | | | |  |
| --- | --- | --- | --- | --- | --- | --- | --- |
| Univariate Moran’s *I* (diagonal) | Physical  proximity | Chemical  pollutants | Hazardous  equipment | Hazardous  conditions | Disease/  infections | Hazardous  noise |  |
| Physical proximity | 0.49 | 0.46 | 0.41 | 0.44 | 0.30 | 0.37 | Bivariate Moran's *I* |
| Chemical pollutants | 0.54 | 0.66 | 0.65 | 0.64 | 0.10 | 0.63 |  |
| Hazardous equipment | 0.47 | 0.98 | 0.64 | 0.63 | 0.04 | 0.63 |  |
| Hazardous conditions | 0.51 | 0.98 | 0.98 | 0.63 | 0.10 | 0.62 |  |
| Disease/infections | 0.45 | -0.10 | -0.20 | -0.11 | 0.30 | 0.03 |  |
| Hazardous noise | 0.43 | 0.97 | 0.95 | 0.95 | -0.18 | 0.65 |  |
|  | Bivariate Correlation (Pearson’s *r*) | | | | |  |  |

| **Supplemental Table S3.** Difference in average nationwide percentile of occupational indicators and the nationwide social vulnerability index (SVI) percentile; values above 0 indicate a higher percentile occupational exposure than the SVI percentile. | | |
| --- | --- | --- |
| **Difference** | **No. tracts (%)** | **Population (%)** |
| -1 to -0.5 | 1,043 (1.4%) | 4,518,847 (1.4%) |
| -0.5 to -0.25 | 10,749 (14.8%) | 47,956,156 (14.9%) |
| -0.25 to -0.05 | 19,418 (26.7%) | 87,961,771 (27.2%) |
| -0.05 to 0.05 | 10,345 (14.2%) | 47,104,175 (14.6%) |
| 0.05 to 0.25 | 18,528 (25.4%) | 83,825,432 (26.0%) |
| 0.25 to 0.5 | 10,707 (14.7%) | 45,943,337 (14.2%) |
| 0.5 to 1 | 1,319 (1.8%) | 4,953,110 (1.5%) |
| Unknown | 727 (1%) | 640,202 (0.2%) |

| **Supplemental Table S4.** Percentage of census tracts in the highest ventile (95-100^th^ percentile) of each occupational indicator by population density, social vulnerability index, racial/ethnic minority percentage, and low-income percentage. | | | | |
| --- | --- | --- | --- | --- |
| **Percent of population racial/ethnic minority** | | | | |
|  | **0 – 25%**  (n = 31,985) | **25 – 50%**  (n = 17,252) | **50 – 75%**  (n = 10,365) | **75 - 100%**  (n = 12,526) |
| Hazardous noise (>20.4%) | 2.6% | 2.4% | 5.8% | 13.6% |
| Hazardous workplace (>52 days) | 2.2% | 2.2% | 6.1% | 14.6% |
| Physical proximity (>104 days) | 2.2% | 2.8% | 4.2% | 11.9% |
| Disease/infection (>33 days) | 3.1% | 3.4% | 3.6% | 7.9% |
|  |  |  |  |  |
| **Percent of population low-income** | | | | |
|  | **0 – 25%**  (n = 28,013) | **25 – 50%**  (n = 31,046) | **50 – 75%**  (n = 11,933) | **75 - 100%**  (n = 1,099) |
| Hazardous noise (>20.4%) | 0.3% | 4.1% | 16.5% | 21.7% |
| Hazardous workplace (>52 days) | 0.4% | 3.8% | 16.8% | 24.3% |
| Physical proximity (>104 days) | 1.0% | 3.6% | 12.5% | 23.7% |
| Disease/infection (>33 days) | 3.7% | 3.7% | 5.4% | 8.6% |
|  |  |  |  |  |
| **Average percent of population racial/ethnic minority and low-income** | | | | |
|  | **0 – 25%**  (n = 29,766) | **25 – 50%**  (n = 24,540) | **50 – 75%**  (n = 13,384) | **75 - 100%**  (n = 4,483) |
| Hazardous noise (>20.4%) | 1.5% | 3.4% | 9.8% | 22.4% |
| Hazardous workplace (>52 days) | 1.3% | 3.0% | 10.3% | 23.4% |
| Physical proximity (>104 days) | 1.4% | 3.3% | 7.9% | 19.7% |
| Disease/infection (>33 days) | 3.1% | 3.4% | 5.8% | 8.6% |
|  |  |  |  |  |

| **Supplemental Table S5.** Census tract-level correlation between occupational hazard indicators and health outcomes | | | |
| --- | --- | --- | --- |
|  | **High BP** | **Asthma** | **Diabetes** |
| Physical proximity | 0.37 | 0.44 | 0.46 |
| Chemical pollutants | 0.44 | 0.42 | 0.55 |
| Hazardous equipment | 0.39 | 0.35 | 0.50 |
| Hazardous conditions | 0.42 | 0.37 | 0.51 |
| Disease/infections | 0.17 | 0.11 | 0.08 |
| Hazardous noise | 0.42 | 0.41 | 0.53 |
| Note: High BP - high blood pressure | | | |


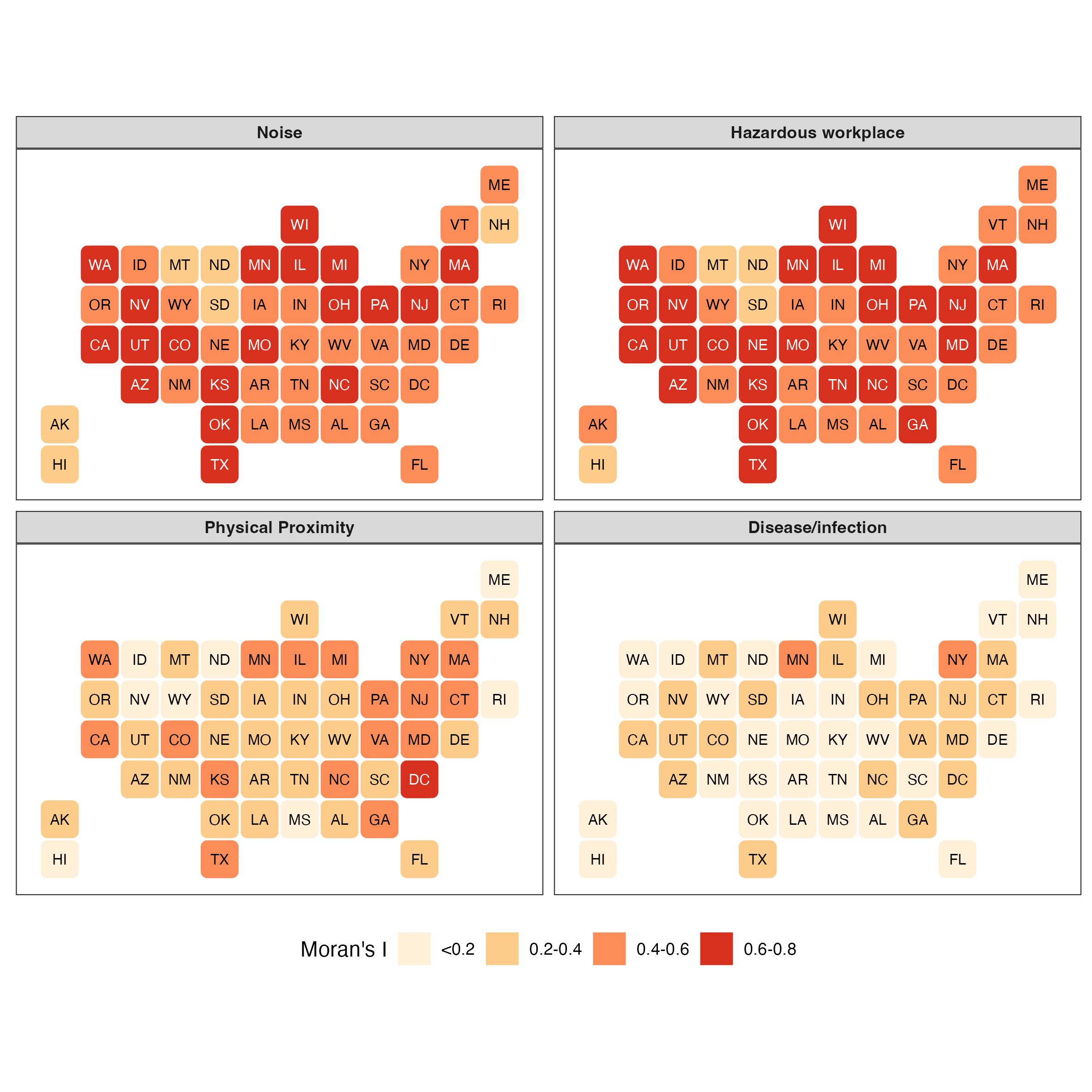
**Supplemental Figure S1.** State-level, univariate spatial autocorrelation (Moran’s *I*) of four occupational indicators, from top to bottom and left to right: percent of workers exposed to hazardous noise (Noise); average days exposed to hazardous workplace conditions, hazardous workplace equipment, and chemical contaminants (Hazardous workplace); average days spent in close physical proximity to other workers (Physical Proximity); and average days exposed to disease/infections (Disease/infection).

| **Supplemental Figure S2.** Local indicators of spatial autocorrelation (LISA, Moran’s *I*) of (A) the prevalence of work-related noise exposure, (B) average days in hazardous workplaces, (C) average days in close physical proximity with other workers, and (D) average days exposed to disease/infection across 72,208 census tracts in the US with working population >20 workers (from left to right, top to bottom) | | | |
| --- | --- | --- | --- |
| **(A) Noise** | | **(B) Hazardous workplace** | |
| 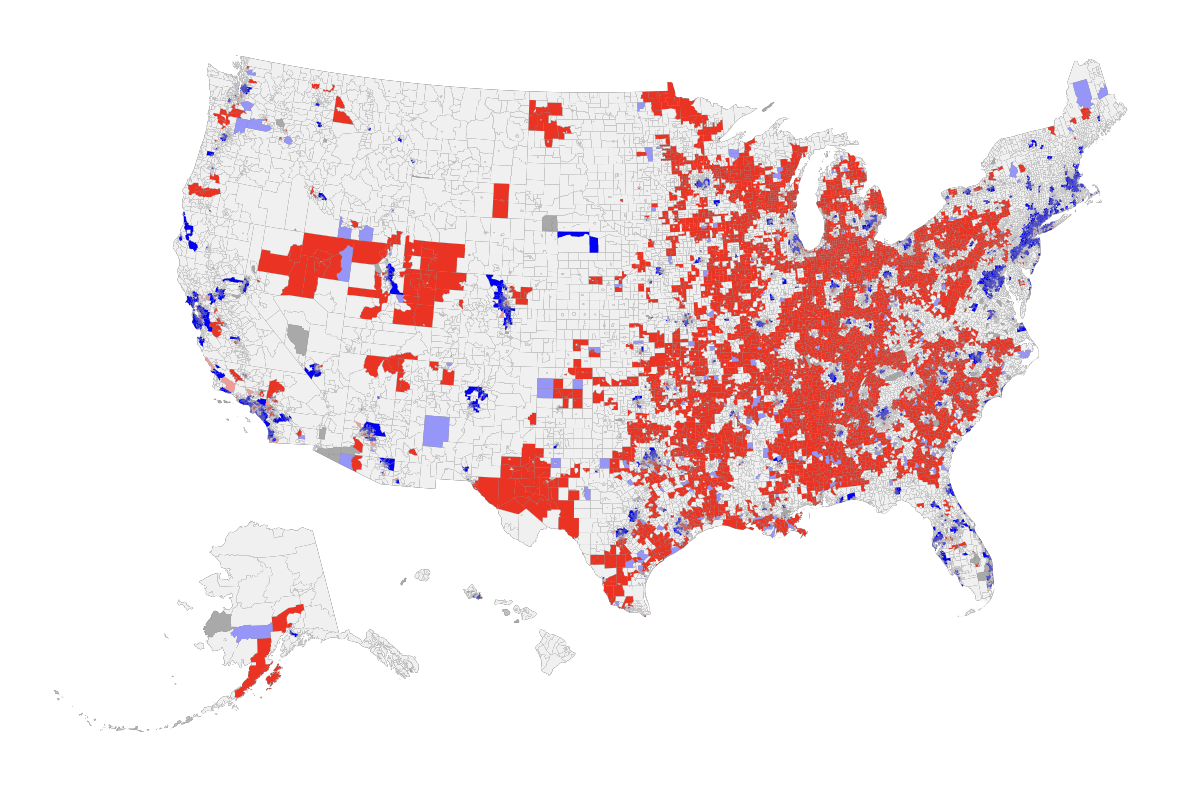 |  | | 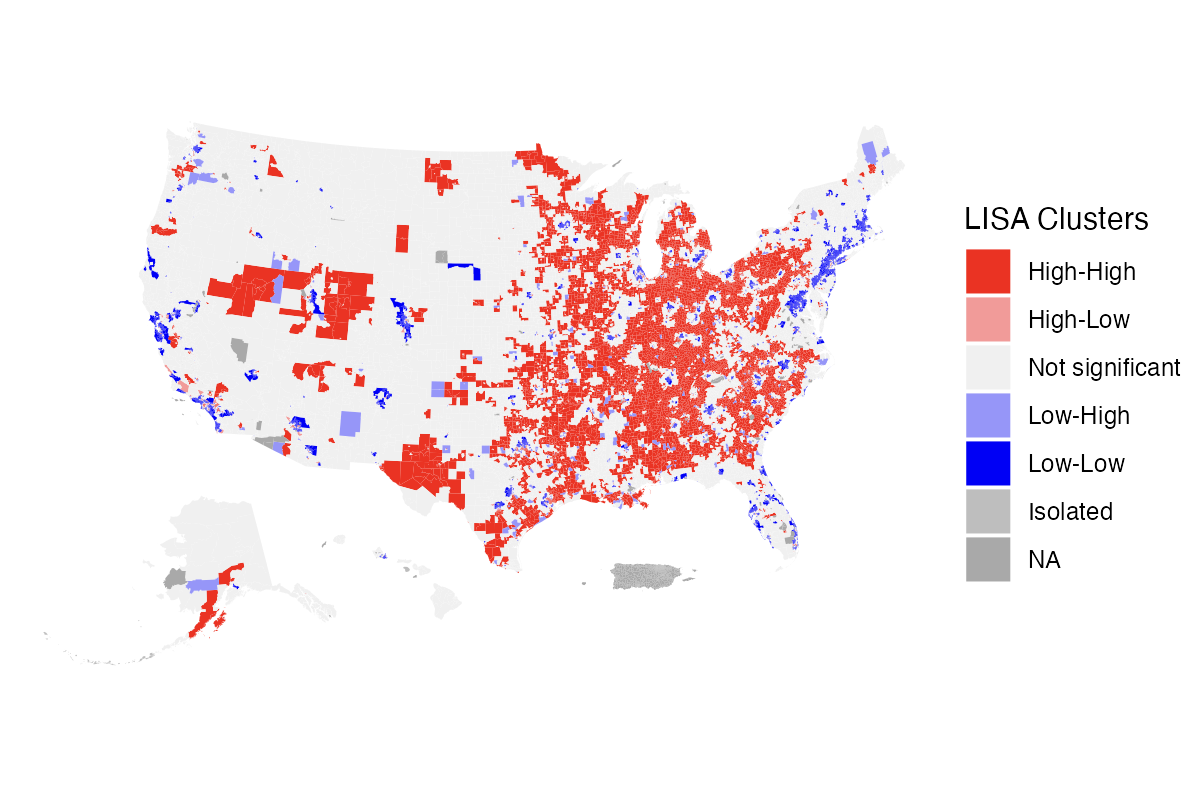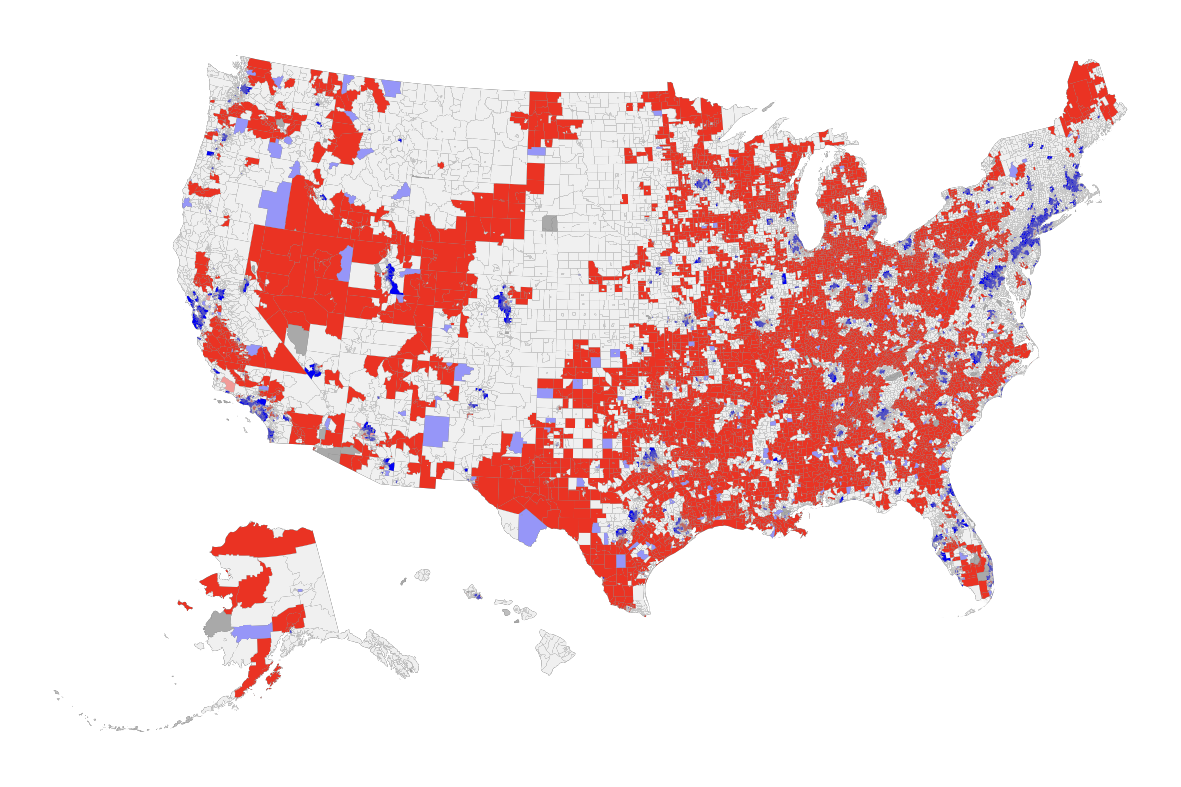 |
| **(C) Physical proximity** | | **(D) Disease/infection** | |
| 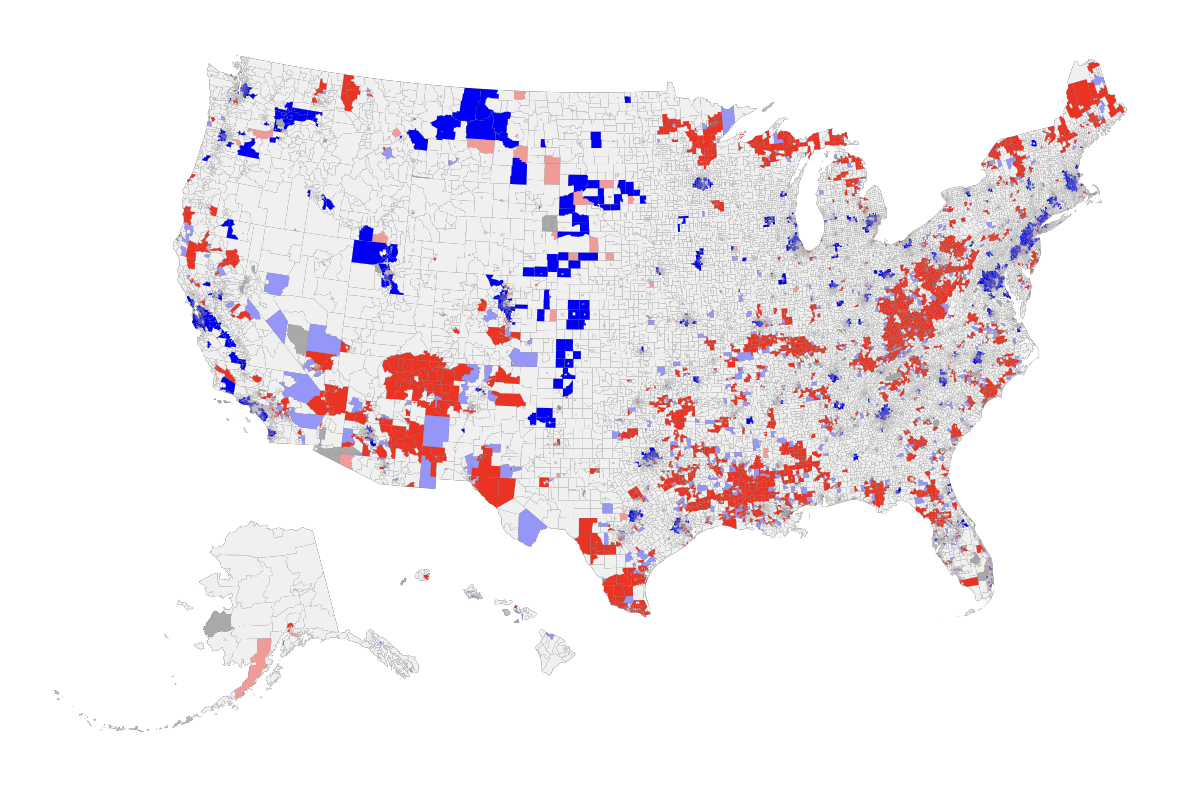 |  | | 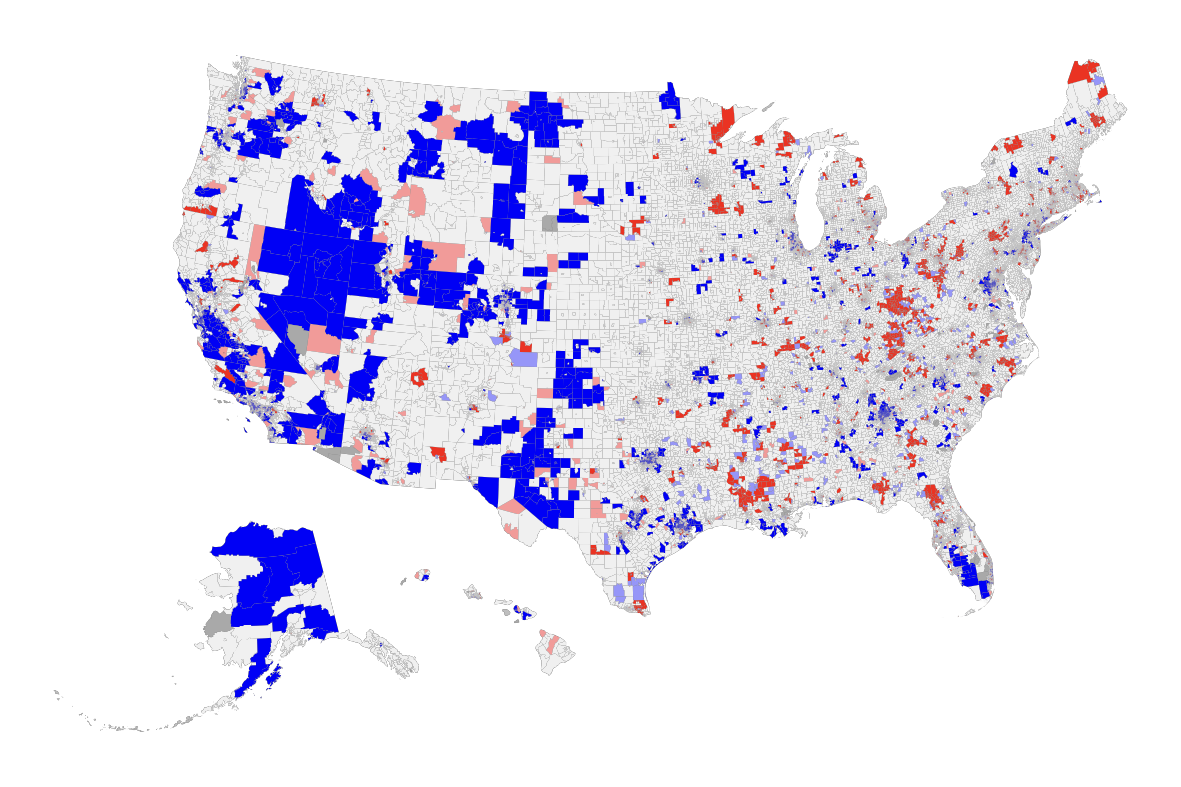 |

| **Supplemental Figure S3.** Bivariate of local indicators of spatial autocorrelation (LISA) of the social vulnerability index (SVI) with the (A) prevalence of work-related noise exposure, (B) average days in hazardous workplaces, (C) average days in close physical proximity with other workers, and (D) average days exposed to disease/infection across 72,208 census tracts in the US with working population >20 workers | | | |
| --- | --- | --- | --- |
| **(A) Noise - SVI** | | **(B) Hazardous workplace - SVI** | |
| 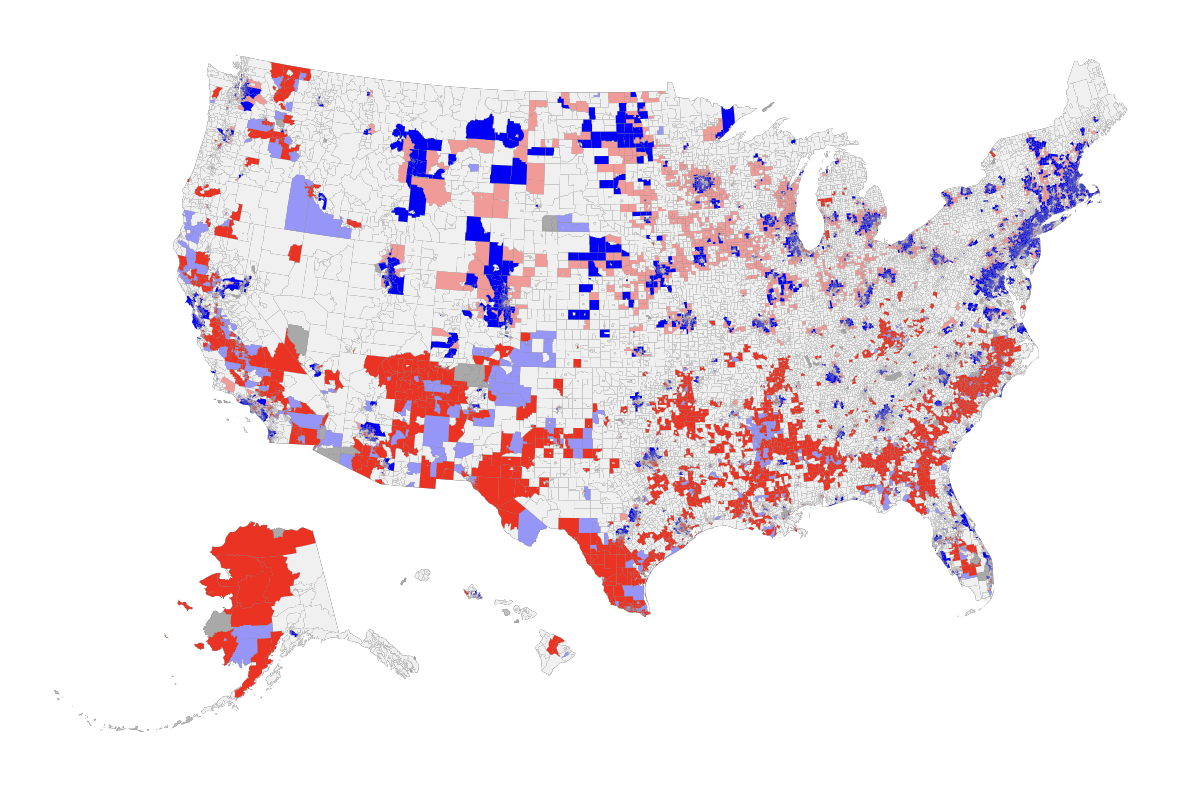 |  | | 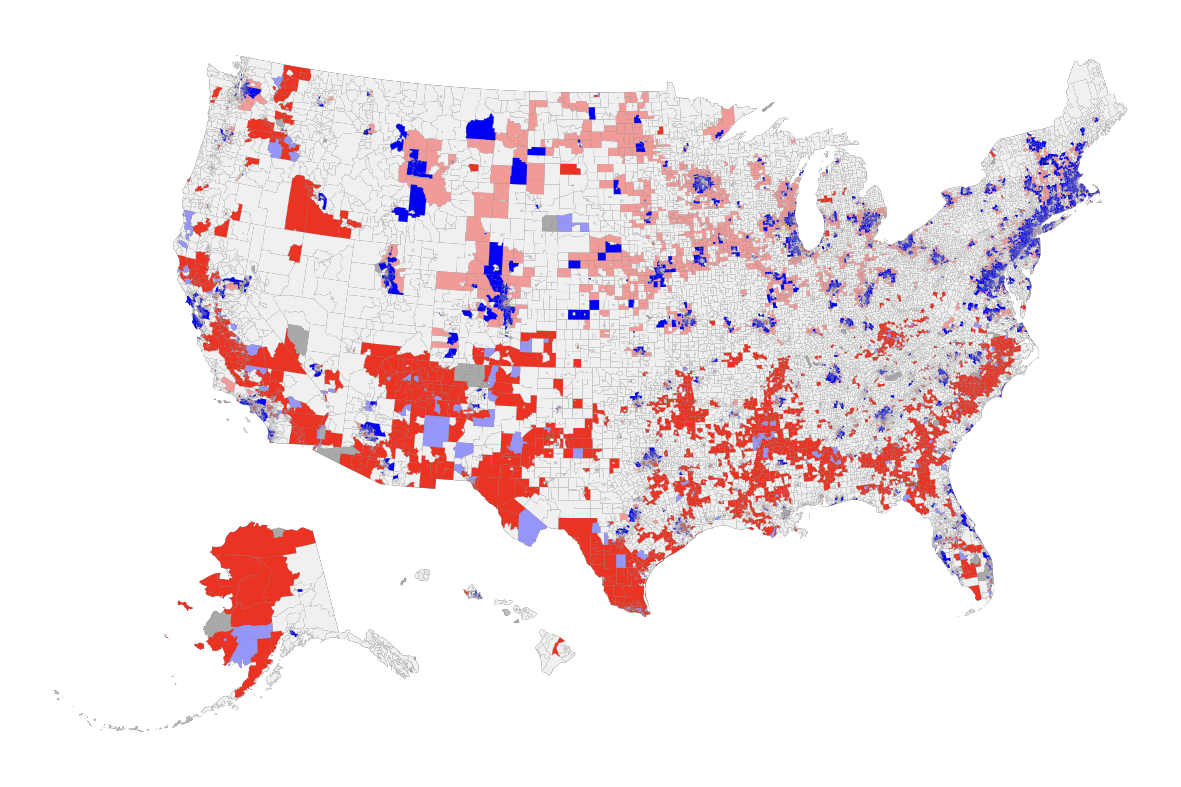 |
| **(C) Physical proximity - SVI** | | **(D) Disease/infection - SVI** | |
| 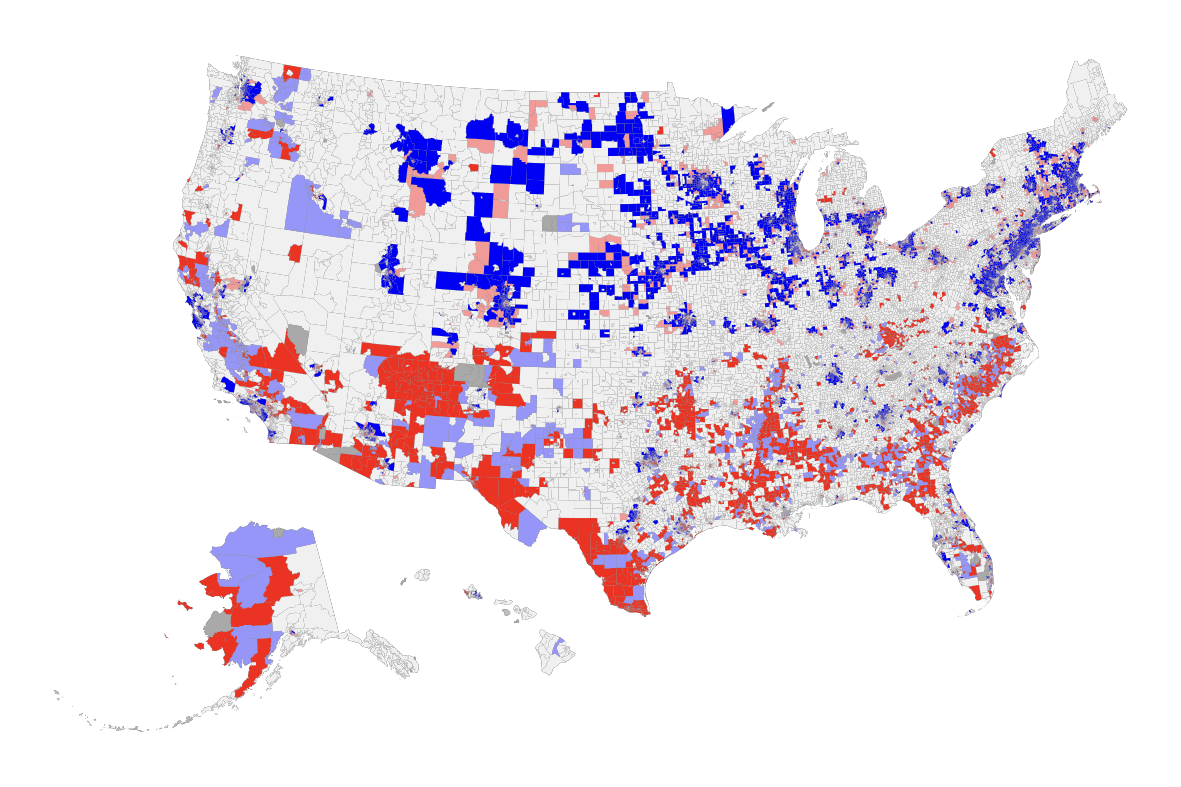 | 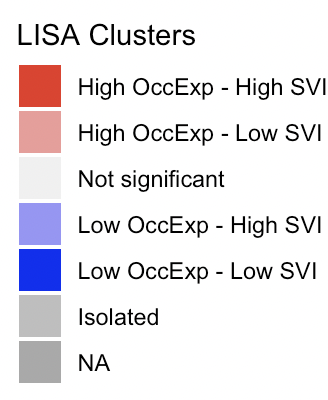 | | 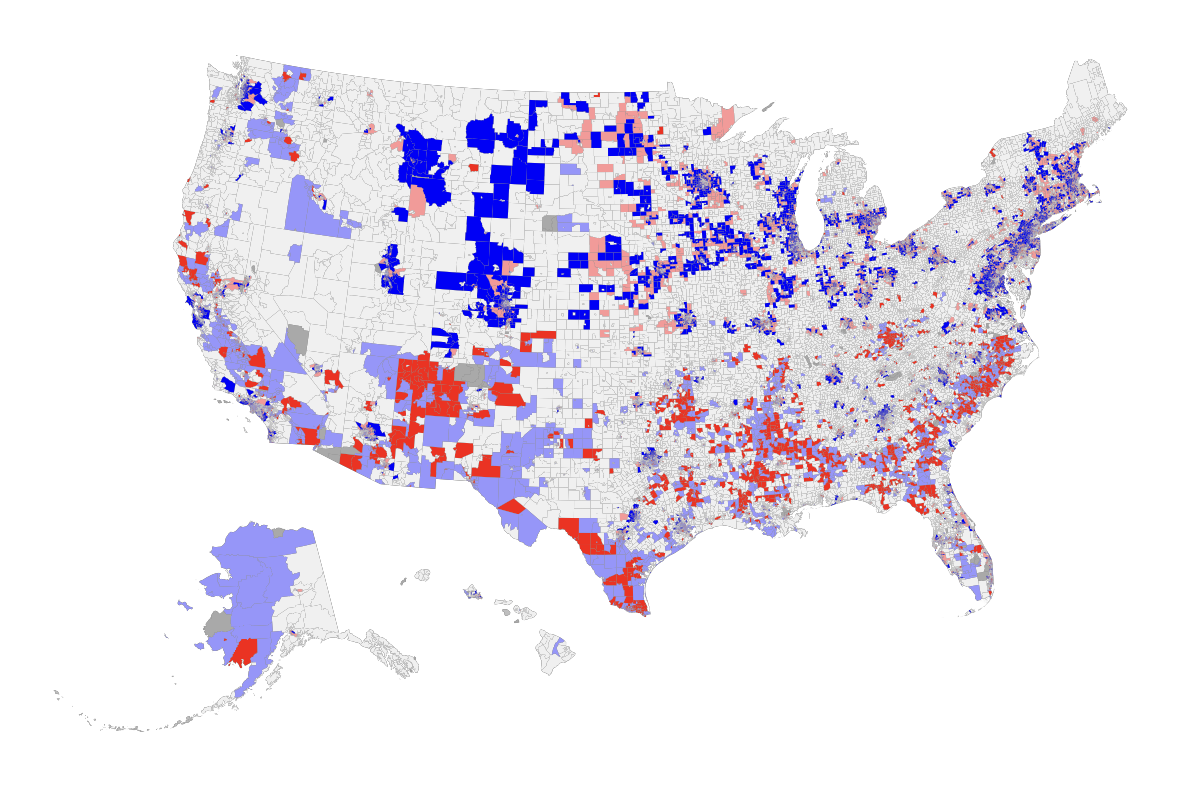 |

**Supplemental Figure S4.** Relationship between the percent of racial/ethnic minorities and the incidence rate ratio (IRR) of (A) diabetes, (B) asthma, and (C) high blood pressure using a natural cubic spline with three degrees of freedom. Each relationship was stratified by high/low noise exposure, chemical pollutant exposure, and disease/infection exposure by splitting census tracts by >50^th^ percentile (high) vs <50^th^ percentile (low) of exposure.


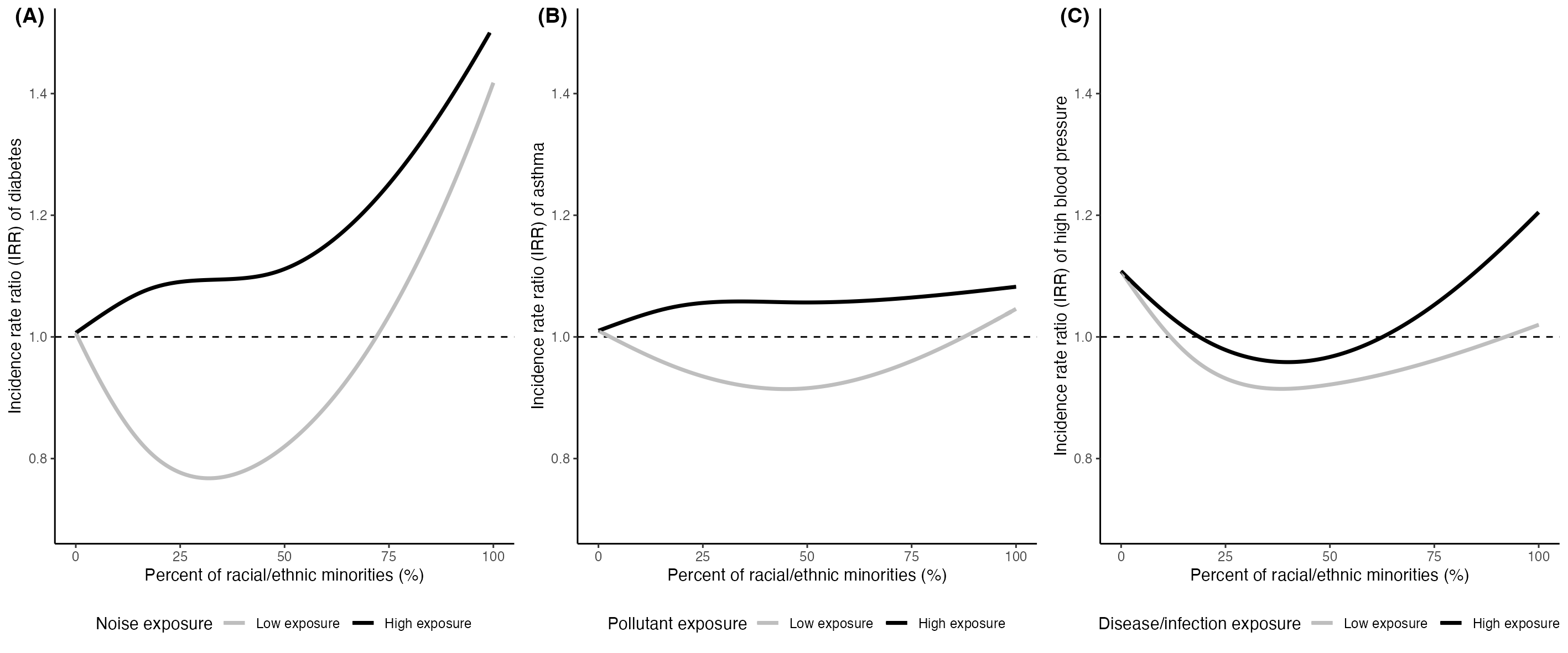


1. Benjamin Roberts et al., “Imputation of Missing Values in a Large Job Exposure Matrix Using Hierarchical Information,” *Journal of Exposure Science & Environmental Epidemiology* 28, no. 6 (2018): 615–48, https://doi.org/10.1038/s41370-018-0037-x. [↑](#footnote-ref-1)
2. American Industrial Hygiene Association Exposure Assessment Strategies Committee, *A Strategy for Assessing and Managing Occupational Exposures*, ed. Steven D. Jahn, William H. Bullock, and Joselito S. Ignacio, 4th ed. (AIHA, 2015). [↑](#footnote-ref-2)
3. Paul Hewett et al., “Rating Exposure Control Using Bayesian Decision Analysis,” *Journal of Occupational and Environmental Hygiene* 3, no. 10 (October 23, 2006): 568–81, https://doi.org/10.1080/15459620600914641. [↑](#footnote-ref-3)
